# Prof. Peto and Sir Richard Doll reply

**Published:** 1986-12

**Authors:** J. Peto, R. Doll


					
Prof. Peto and Sir Richard Doll reply:

Sir - Mr Lee's response to our editorial seems
inappropriate, as we did not discuss the details of
the paper of which he was a co-author (Lee et al.,
1986), and our comments on the interpretation of
his study related solely to inaccurate press coverage
and its exploitation by the tobacco industry. In
relation to the scientific evidence* on passive
smoking, we concluded, in agreement with the
International Agency for Research on Cancer
(1986), that it must be assumed to cause some lung
cancers, but it is impossible to estimate precisely
how many. The evidence, we thought, suggests that
the effect in non-smokers might be to increase the
risk by between about 20% and 50%, which is
consistent with the pooled estimate of about 30%
based on all published studies calculated by Lee et
al. (1986).

There were, however, some differences between
our editorial and the review of the evidence by Lee
et al. (1986). For example, (i) we pointed out that
bias could be in either direction, whereas these
authors mentioned only biases that might increase
the apparent effect of passive smoking; (ii) we
observed that chemical and physical differences
between mainstream and sidestream smoke would
make it impossible to predict the effect of passive
smoking from measurements of, for example,
urinary cotinine even if the form of the dose-
response at very low doses were known; and (iii) we
concluded that the evidence that passive smoking
confers an appreciable risk, although inconclusive,
is suggestive enough to justify concern.

Mr Lee has in his letter consistently selected data
that minimise the predicted risk. He cites the
relative risk of 0.8 from one of several analyses in

Lee et al. (1986), when it might equally cogently be
argued that the relative risk of 1.3 based on cases
whose spouses were interviewed is a more reliable
estimate; he quotes the median urinary cotinine
(6ngm1-1) from a study of non-smokers passively
exposed to smoke (Wald et al., 1984) rather than
the mean, which was over 1 Ing ml-1; he quotes a
3-5 fold increase in lung cancer rates in women
smokers, ignoring evidence that this observed risk is
probably a serious underestimate of the effect of
lifelong smoking in women (Doll et al., 1980); and
he selects particulate retention as a useful index of
exposure in passive smokers, giving a still lower
predicted effect, although there is no evidence that
this is a more appropriate measure of carcinogenic
risk than urinary cotinine.

The most surprising aspect of Mr Lee's letter,
however, is that it should have been addressed to
us at all. The differences between his interpretation
of the evidence and ours are of emphasis rather
than fact, and our principal concern was to draw
attention to the inaccuracy of The Times' report of
his work which was circulated to all MP's by the
Tobacco Advisory Council. We presume that Mr
Lee has privately drawn the Council's attention to
the major factual errors in The Times' report.
Yours etc.

J. Peto,
Section of Epidemiology,
Institute of Cancer Research,
Sutton, Surrey SM2 5PX, UK

and
R. Doll,
Imperial Cancer Research Fund,

Cancer Epidemiology Unit,

Radcliffe Infirmary,
Oxford OX2 6HE, UK.

LETTERS TO THE EDITOR  1021

References

DOLL, R., GRAY, R., HAFNER, B. & PETO, R. (1980).

Mortality  in  relation  to  smoking:   22  years'
observations on female British doctors. Br. Med. J.,
280, 967.

INTERNATIONAL AGENCY FOR RESEARCH ON

CANCER (1986). IARC Monographs on the Evaluation
of the Carcinogenic Risk of Chemicals to Humans. Vol.
38, Tobacco Smoking. International Agency for
Research on Cancer, Lyon.

LEE, P.N., CHAMBERLAIN, J. & ALDERSON, M.R. (1986).

Relationship of passive smoking to risk of lung cancer
and other smoking associated diseases. Br. J. Cancer,
54, 97.

WALD, N.J., BOREHAM, J., BAILEY, A., RITCHIE, C.,

HADDOW, J.E. & KNIGHT, G. (1984). Urinary cotinine
as marker of breathing other people's tobacco smoke,
Lancet, i, 230.

Correspondence on this issue is now closed-Ed.

				


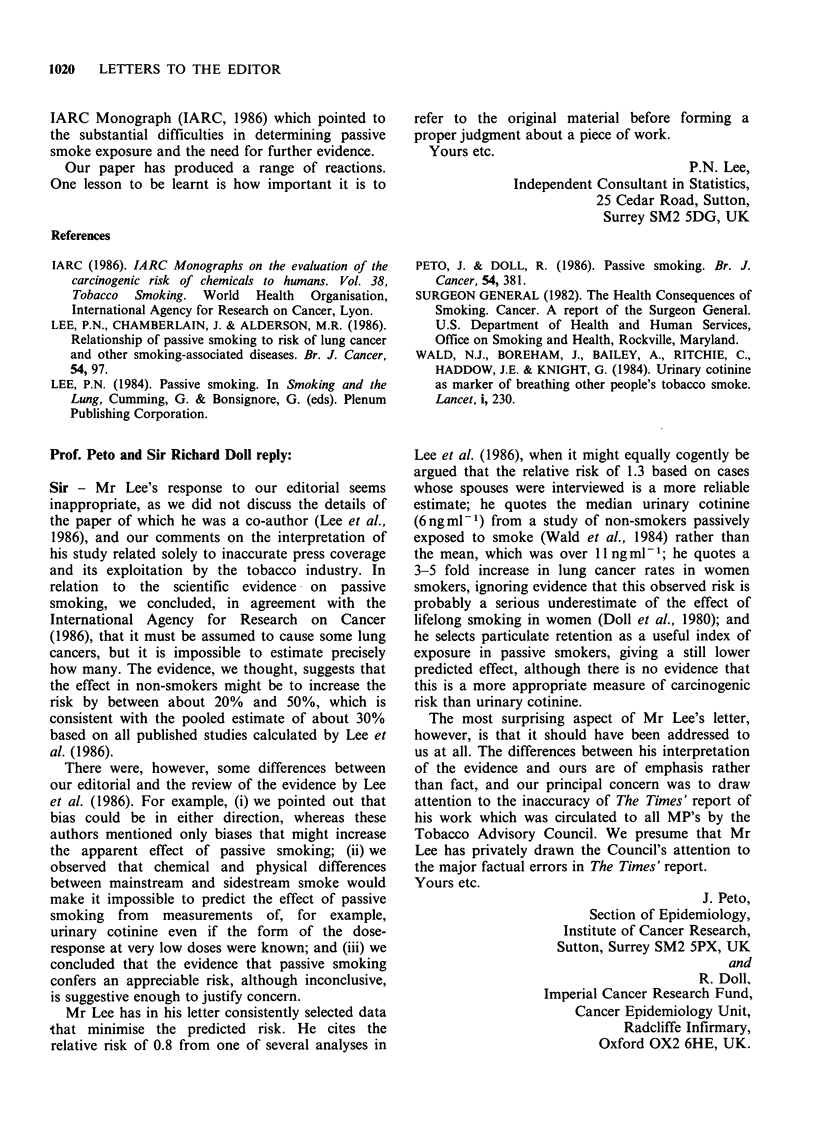

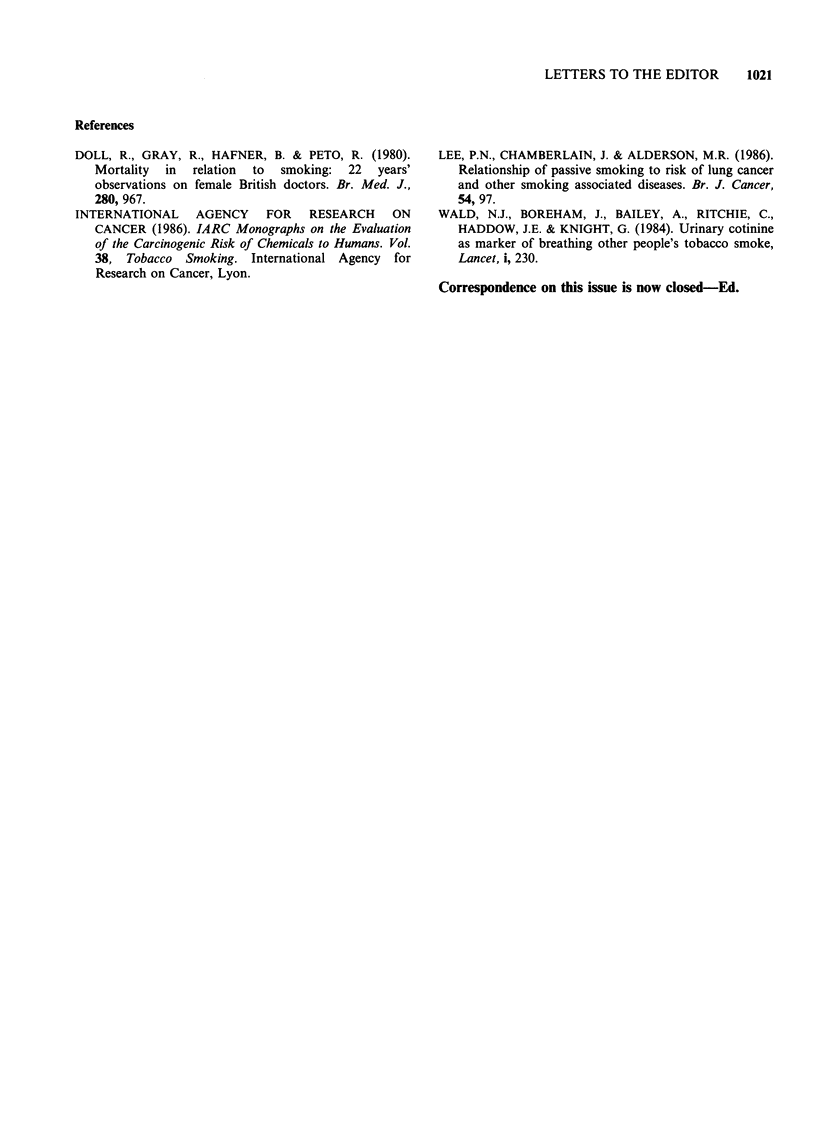

